# Impact of patellar height on unicompartment knee arthroplasty: does patella baja lead to an inferior outcome?

**DOI:** 10.1007/s10195-013-0268-5

**Published:** 2013-09-12

**Authors:** Devdatta Suhas Neogi, Ji Hoon Bae, Chang Woo Seok, Hong Chul Lim

**Affiliations:** Department of Orthopaedic Surgery, Korea University College of Medicine, Guro Hospital, 80 Guro-dong, Guro-gu, Seoul, 152-703 Republic of Korea

**Keywords:** Unicompartmental arthroplasty, Patellar height, Insall-Salvati ratio, Blackburne-Peel Index, Patella baja, UKA

## Abstract

**Background:**

Though a number of series with long-term results have been published, there is still a paucity of literature on the role of patellar height after unicompartment knee arthroplasty (UKA). The present study was conducted with a hypothesis that patella baja may lead to a poor outcome at follow-up.

**Materials and methods:**

A retrospective review of 134 knees was performed and patellar height calculated before and after UKA by Blackburne-Peel index (BPI) and the Insall-Salvati ratio (ISR) on true lateral radiographs of the patients in 30° of flexion taken pre-operatively and at 1 year, 2 years and final follow-up (minimum 5 years). Statistical analysis was performed to evaluate the outcomes.

**Results:**

There was a decrease in ISR in 14.18 % and in BPI in 19.4 % at final follow-up. There was a significant decrease in BPI values while the decrease was not significant for ISR. After eliminating the pre-operative patella baja, 7.3 % developed post-operative patella baja, according to ISR, while 11.5 % developed patella baja as per BPI. At final follow-up there was a statistically significant decrease in stair climbing scores in patients with patella baja when compared to patients with normal ISR.

**Conclusion:**

Patients with a decrease in patellar height as per ISR have a decrease in stair climbing score at mid-term follow-up while the overall KSS, and pain scores are not affected by a change in patellar height and neither is there a significant progress in patellofemoral osteoarthritis among patients with patella baja compared to normal patella.

## Introduction

Unicompartmental knee arthroplasty (UKA) is used as a modality in the management of single compartment arthritis [1–4]. The evolution of patient selection, surgical technique and implant design over the past 30 years has refined the indications and improved the clinical outcome [1–5]. Occurrence of patella infera or baja after total knee arthroplasty has been well reported in the literature, wherein patellofemoral mechanics may be altered, resulting in a decreased post-operative range of motion, extensor lag, anterior knee pain, anterior polyethylene impingement and wear, and diminished outcomes [5–10]. Patella baja can occur in a relative fashion after a TKA because of elevation in joint line or can occur directly because of excessive scar formation, contracture and shortening of the patellar tendon, such as reported after anterior cruciate ligament reconstruction, high tibial osteotomy, fractures about the knee etc. [5–10]. In spite of the surge in literature of results after UKA, the status of patellar height has been infrequently discussed in the literature. Weale et al. [10] reported an infrequent occurrence of patella baja after UKA, while Naal et al. [5] reported that though patella baja occurred with UKA it had no consistent major effect on the clinical outcome at short-term follow-up.

Thus the present study was conducted (1) to assess the patellar height in patients before and after UKA, (2) to see if the amount of pre-operative deformity has any influence on patellar shortening, and (3) to investigate if there is any association between patellar height and clinical outcome at mid-term follow-up. We made a hypothesis that patella baja may lead to a poor outcome at follow-up.

## Materials and methods

The study was performed in accordance with the ethical standards of the 1964 Declaration of Helsinki as revised in 2000. An informed consent form concerning the operative technique to be performed was signed by all patients. Our institutional review board does not require its approval for the review of patient records or images. Patient rights are protected by a law that requires patients to be informed at the time of examination about the possibility that their medical records and radiographs will be reviewed for scientific purposes. A retrospective review was performed for 148 consecutive Oxford medial UKA performed on 122 consecutive patients between June 2001 and June 2005. Informed consent was obtained from all patients. Our indications and selection criteria for surgery were similar to those published in the literature and included non-inflammatory osteoarthritis, symptoms limited to only one compartment, intact anterior cruciate ligament, range of motion of 90° or more, no flexion contracture >5°, no more than a 15° angular deformity [3–5]. Patellofemoral arthritis was considered a contraindication only when the patella-femoral joint osteoarthritis changes were associated with anterior knee pain [1]. Pre-operatively, all patients were examined clinically and radiologically. Radiographic techniques were standardized across all patients and for each radiographic view. A complete set of radiographs of the knee which included antero-posterior (AP), lateral in 30° flexion, standing postero-anterior (PA) in 20° flexion, skyline view of the patella, standing full length scanogram which included hip, knee and ankle and valgus stress radiographs to access the thickness of cartilage in the lateral compartment, were obtained. An MRI scan was obtained when in doubt about the cartilage status. All cases were operated on using a minimally invasive medial para-patellar approach without patellar eversion and Oxford UKA (Biomet Ltd., Bridgend, UK) was implanted in all cases. The details of the operative technique are given in the operative manual [11]. The average hospital stay was 5 days. Patients were encouraged in active movement; isometric quadriceps, straight-leg raising and partial weight-bearing with crutches, as tolerated from the second post-operative day to 4 weeks to allow wound healing and bone adaptation to the new loading pattern.

Patients were called for clinical and radiological follow-up at 6 weeks, 3 months, 6 months, 12 months and yearly thereafter. At follow-up all patients were evaluated with full-length hip–knee–ankle radiographs, AP, lat, PA in 20° flexion standing and skyline of the knee joint. At yearly follow-ups patients were also evaluated clinically using the Knee Society score (KSS) [12] with knee and function subscores, and at final follow-up, a visual analog scale (VAS) (between 0 and 10, with 0 being no pain and 10 the most painful), to assess the presence and intensity of anterior knee pain. Implant failure was defined as conversion to TKA. Ten patients were lost to follow-up. Complete minimum 5-year follow-up data was available for 134 knees in 112 patients for final analysis, of whom we had 30 male and 82 female patients. There were 49 cases with left side, 63 cases with right side and 22 cases with bilateral UKA. Diagnosis was anteromedial arthritis in 132 knees, and osteonecrosis and post traumatic arthritis in one knee each. Average age at surgery was 64.2 years (range 51–74) and mean body mass index was 28.2 (range 19.6–37.8). The average follow-up was 6.4 years (range 5.1–8.1 years).

Patellar height was determined using the Blackburne-Peel index (BPI) [13] and the Insall-Salvati ratio (ISR) [14] on true lateral radiographs of the patients in 30° of flexion taken pre-operatively and at 1 year, 2 years and final follow-up (minimum 5 years) was determined independently by two observers and their mean was used to calculate for statistical analysis. Since it is difficult to precisely define the point of origin and insertion of the tendon, and since the shape of the patella in some cases may change after the removal of osteophytes, the three radiographs of the patient were reviewed simultaneously but independently by both the observers. The same points were identified on each and measurements were made between a point on the inferior pole of the patella and the tibial tuberosity. The ISR was determined by dividing the length of the patellar tendon (measured as previously stated) by the longest dimension of the patella on the lateral X-ray. The BPI was determined by dividing the distance from inferior tip of distal patellar articular surface to a tangent to the tibial articular surface. The measurement was recorded to the nearest 0.1 mm using the digitized computer system STAR PACS Pi view Star 5.0.6.1 software (INFINITT technology 2004, Seoul, South Korea), and the magnification factor (10 %) was corrected automatically in the program. To classify patients, we considered an ISR < 0.8 or BPI < 0.5 as patella baja, an ISR 0.8–1.2 or BPI 0.5–1.0 as normal and an ISR > 1.2 or BPI > 1.0 as patella alta [13, 14]. The mechanical varus valgus alignment was determined using full-length radiographs. Five-year follow-up radiographs were assessed and compared with the pre-operative radiographs, looking for progression of osteoarthritis in the patellofemoral joint, which was classified as per the Kellgren and Lawrence (KL) classification [15] as the presence and extent of radiolucency and evidence of component subsidence. To define the varus or valgus alignment we used a mechanical tibial femoral angle which was formed by the line joining the center of the femoral head to the center of the knee joint and a second line from the center of the knee joint to center of the ankle joint. The angle was measured on the lateral side and a value of more than 180° implied a varus alignment and <180° a valgus alignment. The mechanical axis deviation (MAD) was measured pre-operatively and at the most recent assessment using standing, long-alignment radiographs to determine the location of the mechanical axis with respect to the center of the tibial surface, as described by Kennedy and White. Zones 1 and 2 are on the medial side of the tibial eminence, and zones 3 and 4 are on the lateral side of the tibial eminence. Zone C is the central part of the tibial plateau [16, 17]. It was our aim to obtain the MAD preferably in zone C or 2. We preferred a tibial slope of 0° for the tibial implant. For the purposes of clarity, a varus angle is positive, and any valgus angle is negative. The tibial resection angle was measured from the anteroposterior standing film. This is defined as the difference between pre-operative medial proximal tibial angle (MPTA) and post-operative MPTA (angle between the long axis of the tibia and the superior edge of the tibial insert) [17]. Varus and valgus alignment of the femoral component was measured against the long axis of the tibia. Varus was defined as a medial inclination of the proximal part of the femoral profile [17].

Statistical analysis was used to determine the effect that UKA surgery had on the ISR and BPI. Interobserver variability was assessed by the intraclass correlation coefficient (ICC) alpha. Paired *t*-tests were used to compare patellar ratios where specific differences occurred. The linear regression analysis was used to analyze the effect of the measured ISR and BPI on the post-operative variables of range of motion, KSSs, and functional score. Log linear regression analysis was used to evaluate the effect of the ISR and BPI on post-operative pain scores and the ability to climb stairs as determined by the Knee Society rating system. Statistical analysis was performed using the software package SPSS version 14 (SPSS Inc., Chicago, IL, USA).

## Results

The mean follow-up was 6.2 years (range 5.2–8 years). The ICC was 0.876 (95 % CI 0.766–0.903) for pre-operative and 0.832 (95 % CI 0.734–0.910) for post-operative ISR measurements while it was 0.796 (95 % CI 0.743–0.872) for pre-operative and 0.864 (95 % CI 0.810–0.912) for BPI. The pre-operative and postoperative ISR and BPI values classified as normal, patella baja and patella alta are presented in Table 1. There was a significant decrease in the BPI values from a pre-operative value of 0.748 (SD ± 0.122) to 0.716 (SD ± 0.110) at 1 year after surgery (*p* = 0.0051), while there was not a significant change to 0.711(SD ± 0.114) at final follow-up (>5 years) (*p* = 0.137). There was no significant decrease in ISR from a value of 0.981(SD ± 0.129) to 0.974 (SD ± 0.135) at 1 year and 0.971(SD ± 0.139) at final follow-up (>5 years) (*p* > 0.05). When calculated irrespective of the pre-operative classification, patella baja was found in 14.18 % of patients as per the ISR and 19.4 as per BPI. After eliminating the pre-operative patella baja, 7.44 % (9/124 knees with normal or patella alta) developed post-operative patella baja according to ISR while 11.5 % developed patella baja as per BPI. The average length of patellar tendon pre-operatively was 44.13 mm and at final follow-up it was 43.26 9 (*p* = 0.348) (Table 2).

**Table 1:** Classification of patients as per patellar height indices

Classification	Post-op baja	Post-op normal	Post-op alta	Total
Classification of patients by Insall-Salvati Ratio (ISR)				
Pre-op baja	10	0	0	10
Pre-op normal	9	112	0	121
Pre-op alta	0	0	3	3
Total	19	112	3	134
Classification of patients by Blackburne-Peel Index (BPI)				
Pre-op baja	12	0	0	12
Pre-op normal	14	108	0	122
Pre-op alta	0	0	0	0
Total	26	108	0	134

Classification of ISR < 0.8 or BPI < 0.5 is patella baja, an ISR 0.8–1.2 or BPI 0.5–1.0 is normal and an ISR > 1.2 or BPI > 1.0 is patella alta

**Table 2:** Patellar tendon length over time

	Pre-op	1 year follow-up	2 year follow-up	Final follow-up	*p* (between pre-op and final follow-up)
Over-all	44.13 (SD ± 8.14)	43.022 (SD ± 8.32)	43.058 (SD ± 8.1)	43.26 (SD ± 0.79)	0.348
Pre-op patella baja persisting as post–op patella baja according to ISR	35.16 (SD ± 3.5)	34.54 (SD ± 3.7)	34.68 (SD ± 3.66)	34.45 (SD ± 3.54)	0.0861
Pre-op normal patellas which became post-op patella bajas	38.11 (SD ± 4.3)	35.58 (SD ± 3.9)	35.68 (SD ± 4.2)	35.11 (SD ± 4.1)	0.0065

All measurements in mm

**Fig. 1:**
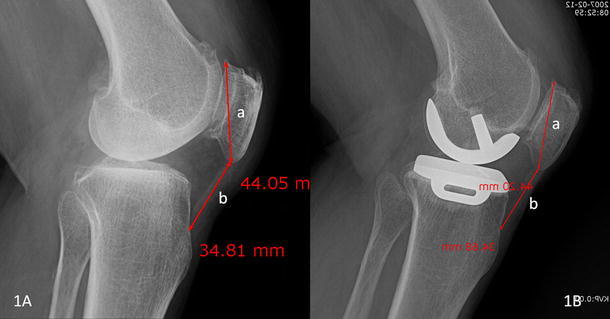
**a** Pre-operative Blackburne-Peel index: a normal result, as measured by b/a, is 0.562. **b** A post-operative Blackburne-Peel index of 0.487 as measured by b/a, is classified as patella alta

**Fig. 2:**
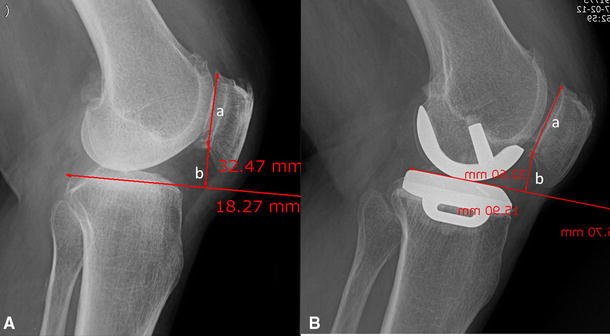
**a** Pre-operative Insall-Salvati ratio: a normal result, as measured by b/a, is 0. (Same as case in Fig. 1). **b** A post-operative Insall-Salvati ratio, as measured by b/a, is 0.79 and is normal

When the effect of ISR and BPI among different groups classified as patella baja, normal patella and patella alta was compared on the outcomes of KSS, functional score, stair climbing, range of motion and pain, no statistically significant differences were seen between either groups at 2 years, while there was a statistically significant decrease in stair climbing scores (*p* = 0.012) in patients with patella baja when compared to patients with normal ISR at final follow-up (Figs. 1, 2).

Though there was a decrease in overall function score at 5 years, there was no statistically significant difference between patients with patella baja, normal patella and patella alta with both BPI and ISR. Decrease in post-operative ISR and BPI did not co-relate with decrease in knee score (*r* = 0.121), postoperative range of motion (*r* = 0.027) and pain scores (*r* = −0.0213) during follow-up.

We evaluated the change in the status of patellofemoral arthritis at final follow-up and there was not much change in grade as per KL classification (Table 3). However, we had seven cases that showed mild to moderate anterior knee pain by the visual analog scale on walking and three among these cases had patella baja post-operatively by either or both ISR. These cases pre-operatively had grade II arthritis as per KL classification. When the pre-operative mechanical tibio-femoral angle was compared between different groups, there was no significant difference seen between them (Table 4). The position of the implant did not affect the development of patella baja (Table 5).

**Table 3:** Progression of osteoarthritis at final follow-up as per Kellgren–Lawrence (KL) classification

Type of PB	Pre-operative KL grade	Final follow-up KL grade	Anterior knee pain at final follow-up
All cases	GI-56	GI-42	VAS 5–2 cases
	GII-69	GII-76	VAS 3–4 cases
	GIII-9	GIII-12	VAS 7–1
	GIV-0	GIV-4	
PB pre-op and PB post-op (10 cases)	GI-4	GI-2	VAS 5–1 case
	GII-5	GII-5	VAS 3–1
	GIII-1	GIII-2	
	GIV-0	GIV-1	
NP pre-op, PB post-op (9 cases)	GI-1	GI-0	VAS 3–1
	GII-7	GII-7	
	GIII-1	GIII-2	
	GIV-0	GIV-0	
PB post-op irrespective of pre-op status (19 cases)	GI-5	GI-2	VAS 5–1 case
	GII-12	GII-12	VAS 3–2
	GIII-2	GIII-4	
	GIV-0	GIV-1	

*p* > 0.05

*PB* patella baja,*NP* normal patella

**Table 4:** Relation of mechanical tibio-femoral angle (MTFA) and patella baja

	Pre-op MTFA	*p* value when compared with overall pre-op MTFA	Post-op MTFA
Over-all	188.34° (SD ± 6°)	–	182.4° (SD ± 3.8°)
Pre-op patella baja persisting as post-op patella baja according to ISR	187 (SD ± 4°)	0.162	184.6° (SD ± 3°)
Pre-op normal patella which became post-op patella baja	188.58 (SD ± 4°)	0.082	185.2° (SD ± 3.2°)

*p* > 0.05 when compared between groups

**Table 5:** Relation between implant positioning and patellar indices

	Normal patellar indices	Patella baja	*p* value
Femoral inclination	0.5° (SD ± 2°)	0.4° (SD ± 2°)	0.11
Tibial cut angle	+0.45° (SD ± 3.2°)	+0.5° (SD ± 2.8°)	0.093

+ indicates varus and − indicates valgus

We had two cases of recurrent dislocation of the polyethylene insert which were revised to TKA. Both the cases had a normal ISR and BPI index. In the first case the insert dislocation occurred at 3 months, and a thicker insert was substituted. However, it dislocated again and ultimately was revised to TKA. The second case showed insert dislocation at 6 years. Since there was grade IV OA in the patellofemoral joint and lateral compartment as per KL classification, and also associated anterior knee pain, this second case was revised to TKA.

## Discussion

Though a number of series have been published documenting good results after UKA, there has been a paucity of literature on the role of UKA on the length of the patella tendon or the incidence of patella baja after UKA [1–4, 18]. In spite of indications for this procedure in unicompartmental arthritis having been established, there has been some controversy regarding the status of the patellofemoral joint [1, 2, 4, 5]. A recent study [5] had concluded that patellar height had no consistent major effects on early clinical outcome after UKA, and hence the patellar height might not be considered as a strict separate patient-selection criterion. The purpose of this study was to evaluate the patients for a change in patellar height by ISR and BPI after UKA and to see if there is a difference in clinical and radiological outcomes for these patients. Before the study we had assumed that at mid-term follow-up a progression in patellofemoral arthritis may lead to progressive impingement, anterior knee pain and adversely affect the outcome in patients who develop patella baja post-operatively. However, our results showed a significant decrease in only the stair-climbing component of the functional aspect of KSS for patients with patella baja assessed with ISR while this decrease was not significant in patients with patella baja assessed with BPI, and hence we had to reject our previous hypothesis.

Patella baja refers to an inferior position of the patella in the sagittal plane and can be determined with numerous radiographic measurements [13, 14, 19]. Most involve a ratio between a measure of patellar length and a measure of the distance between an aspect of the patella and a landmark on the tibia. The use of a ratio rather than an absolute length compensates for variations in patients’ height. We used both the ISR and BPI to measure the patellar height after UKA. The BPI takes the joint-line into consideration. In contrast, the ISR ignores the joint-line and relates more to the patellar-tendon length. Grelsamer [7] described a decrease in BPI values and non-decreased ISR values to indicate an elevation of the joint-line, defined as pseudo-patella baja. However, an elevation of the joint-line is hardly possible after UKA, since the contralateral compartment is not affected by the surgery [5]. It may nevertheless be that UKA created a “step” in the joint-line from the medial to the lateral compartment, and referring to the upper polyethylene border for the measurements, BP values might be decreased by such a step [5]. Finally, BPI values might have been decreased by a true decrease of the patellar height. The decrease in patellar height after TKR has been reported in more than 50–64 % [8, 9] of cases and patella baja develops in 9–25 % of patients without lateral release, and all patients with patellar release [9, 10]. Weale et al. [10] reported that there was no significant change in patellar height after UKA at 8 months or 5 years after surgery, while Naal et al. [5] did observe a significant decrease in patellar height as per BPI and no significant decrease as per ISR at short-term follow-up. Our results at mid-term follow-up are also in agreement with these results of Naal et al. [5] which they observed at short-term follow-up. Also, in contrast to a study by Weale et al. [10] we observed an overall decrease in patellar tendon length and that this change was not progressive over the follow-up period. In contrast to TKA, fewer patients develop patella baja after UKA and this may be due to the minimally invasive incision, avoiding patellar eversion and minimal trauma to Hoffas’ fat pad and extensor mechanism. Much of the literature concerning UKA has advocated relative under-correction of the alignment of the knee with the presumption that overcorrection increases the risk of failure by progression of arthritic change of the lateral compartment [20–22]. Scott et al. [23] reported that overcorrection of alignment increases the risk of degenerative change in the contralateral compartment. However, progression of arthritis in the lateral compartment could be simply caused by the natural progression of the underlying arthritic disease [24]. Barrett and Scott [25] reported that under-correction of alignment increases the wear of polyethylene implants and the recurrence of the deformity. Kennedy and White [16] reported that the best result can be obtained when the mechanical axis runs through the center of the knee joint or slightly medial from the center of knee joint, and we aimed for the same in our cases. The position of the implant did not affect the development of patella baja. This may be because limited exposure and careful soft tissue resection prevented damage to the patellar tendon. In spite of a small incision, it was observed that a large proportion of implants were placed in an acceptable position. Moreover, as UKA involves only the medial compartment, it does not effectively change the position of the joint line [17].

Meneghini et al. [9] studied over 1,000 patients with TKA and concluded that a decrease in patella tendon length occurs; stair and function scores after TKA are adversely affected compared with patients in whom the ISR is not decreased. Other studies have associated patella infera after TKA with decreased post-operative range of motion, extensor lag, anterior knee pain, anterior polyethylene impingement and wear, and diminished outcomes [5–9]. In contrast, after UKA no consistent major effects on early clinical outcome were seen and a weak negative correlation between lower pre-operative BP values and the post-operative knee extension, and a weak negative correlation between lower pre-operative IS values and post-operative knee scores were seen [5]. Our results did show a significant difference only in stair-climbing scores at final follow-up for patients with post-operative patellar baja. However, when the scores were compared between those patients who had pre-operative patella baja according to both ISR and BPI, who became normal after surgery, and those who remained patella baja after surgery, there was no significant difference between them. Thus, it shows that a corrective effect of surgery on pre-operative patellar baja is not present.

The current study shows a decline in functional scores over time in patients undergoing UKA. However, this decrease did not correlate with patellar height. The decrease in functional status may be related to the increase in age of the patients, considering that the mean age at which the patients underwent UKA in our study was 64.2 years.

Progression of patellofemoral arthrosis after UKA is a possibility and Berger et al. [1] observed patellofemoral symptoms were present in 1.6 % of patients at 10 years which increased markedly to 10 % of patients at 15 years. Hernigou and Deschamps [26] reported that after UKA the patellofemoral joint was affected by degenerative changes and patellar impingement against the femoral component and that both factors negatively influenced the functional outcome. Patellar impingement with the Oxford UKA prosthesis, as in our study, may not be a problem as during the surgical procedure care is taken to remove the bone anteriorly until there is at least 4 mm clearance for the front of the bearing in full extension. Price et al. [4] ignored the state of the patellofemoral joint in the absence of anterior knee pain at surgery and at 10 years follow-up; 97 % of knees reviewed had no patella-femoral pain and also the main cause for revision in their series was progression of osteoarthritis in the lateral compartment. At an average follow-up of 6.4 years we had 5.22 % of patients who complained of anterior knee pain. However, in only one patient with associated anterior knee pain was a TKA performed.

We do have limitations in this study, in that it is a retrospective review of cases. However, there are many strengths like use of a single implant, a single surgeon being the main operating surgeon, medium duration of follow-up and a sufficiently large number of cases: all of which may offset the study limitation.

In summary, this study documents a decrease in patellar height after UKA by both ISR and BPI. In this study, 7.3 % developed post-operative patella baja according to ISR while 7.8 % developed patella baja as per BPI. Patients with a decrease in patellar height as per ISR have a decrease in stair-climbing score at mid-term follow-up. There was no effect on the correction of pre-operative patella baja to normal post-operatively. The overall KSS and pain score are not affected by a change in patellar height and neither is there a significant progress in patellofemoral osteoarthritis among patients with patella baja compared to normal patella.
